# Prediction Model of HBsAg Seroclearance in Patients with Chronic HBV Infection

**DOI:** 10.1155/2020/6820179

**Published:** 2020-08-14

**Authors:** Jing Cao, Jiao Gong, Christ-Jonathan Tsia Hin Fong, Cuicui Xiao, Guoli Lin, Xiangyong Li, Yusheng Jie, Yutian Chong

**Affiliations:** ^1^Department of Infectious Diseases, The Third Affiliated Hospital of Sun Yat-sen University, Guangzhou, 510630 Guangdong Province, China; ^2^Department of Laboratory Medicine, The Third Affiliated Hospital of Sun Yat-sen University, Guangzhou, 510630 Guangdong Province, China; ^3^Department of Gastroenterology, The Third Affiliated Hospital of Sun Yat-sen University, Guangzhou, 510630 Guangdong Province, China; ^4^Department of Anesthesiology, The Third Affiliated Hospital of Sun Yat-sen University, Guangzhou, 510630 Guangdong Province, China

## Abstract

**Background:**

Prediction of HBsAg seroclearance, defined as the loss of circulating HBsAg with or without development of antibodies for HBsAg in patients with chronic hepatitis B (CHB), is highly difficult and challenging due to its low incidence. This study is aimed at developing and validating a nomogram for prediction of HBsAg loss in CHB patients.

**Methods:**

We analyzed a total of 1398 patients with CHB. Two-thirds of the patients were randomly assigned to the training set (*n* = 918), and one-third were assigned to the validation set (*n* = 480). Univariate and multivariate analysis by Cox regression analysis was performed using the training set, and the nomogram was constructed. Discrimination and calibration were performed using the training set and validation set.

**Results:**

On multivariate analysis of the training set, independent factors for HBsAg loss including BMI, HBeAg status, HBsAg titer (quantitative HBsAg), and baseline hepatitis B virus (HBV) DNA level were incorporated into the nomogram. The HBsAg seroclearance calibration curve showed an optimal agreement between predictions by the nomogram and actual observation. The concordance index (C-index) of nomogram was 0.913, with confirmation in the validation set where the C-index was 0.886.

**Conclusions:**

We established and validated a novel nomogram that can individually predict HBsAg seroclearance and non-seroclearance for CHB patients, which is clinically unprecedented. This practical prognostic model may help clinicians in decision-making and design of clinical studies.

## 1. Introduction

HBV infection continues to be a global health problem. Worldwide, around 2 billion people have evidence of past or present infection with HBV and an estimated 257 million are chronically infected [1]. Almost half of the world's population resides in areas of high HBV endemicity, with the highest prevalence in Africa and East Asia. In addition, in China, approximately 300 million people suffer from hepatopathy, having a major impact on the global burden of liver diseases [2]. Patients with chronic HBV infection have an increased risk of developing sequelae such as cirrhosis and hepatocellular carcinoma (HCC). Chronic infection is characterized by the persistence of hepatitis B virus surface antigen (HBsAg) for at least 6 months (with or without concurrent hepatitis B virus e-antigen (HBeAg)). Persistence of HBsAg is a surrogate marker for the risk of developing chronic liver disease and HCC. Recent studies have focused on the role of HBsAg quantification in seroclearance of HBsAg which usually indicates that HBV infection has been cured [3].

Nomograms are widely used as prognostic devices in medicine, especially for individualized estimation of cancer survival. With the ability to generate the probability of a clinical event by integrating diverse prognostic and determinant variables, nomograms can meet our need for biologically and clinically integrated models and fulfill our drive towards personalized medicine [4]. Current evidence suggests that the occurrence of HBsAg seroclearance in patients with chronic HBV infection is a rare event that occurs at 1% to 2% per year, usually after a long duration of sustained biochemical remission [5], and its probability to be forecasted is rarely known. In this study, we sought to develop a clinical nomogram for predicting the rate of HBsAg loss of patients with CHB.

## 2. Methods

### 2.1. Study Design and Patients

From 2009.1 to 2018.6, a total of 3220 patients were diagnosed with CHB and were followed up every 3 to 6 months by the Infectious Diseases Department of The Third Affiliated Hospital of Sun Yat-sen University, Guangzhou, China. All patients had been HBsAg positive for more than 6 months and were excluded for at least one of the following conditions: lost to follow-up for over 12 months, presence of comorbidities (hepatitis A/C/E virus coinfection, autoimmune liver diseases, other malignant tumors, renal insufficiency, hepatolenticular degeneration, and alcoholic liver disease), having received immunosuppressive (transplantation) therapy, and cases of data loss. All enrolled patients signed informed consent.

### 2.2. Variables and Data Collection

We recorded the following data for each patient: gender, age, body mass index (BMI), alcohol history, family history, diagnosis, treatment, and other laboratory indexes, such as alanine aminotransferase (ALT), aspartate aminotransferase (AST), total bilirubin, and albumin (ALB). The reference ranges of the biochemical index are as follows: ALT: 3–35 U/l; AST: 15–40 U/l; total bilirubin: 4.0–23.9 *μ*mol/l; and ALB: 36.0–51.0 g/l. Furthermore, we also collected the following serological and virological markers: hepatitis B virus surface antigen (HBsAg), hepatitis B virus surface antibody (HBsAb), hepatitis B virus e-antigen (HBeAg), hepatitis B virus E antibody (HBeAb), and hepatitis B virus core antibody (HBcAb), measured by chemiluminescence immunoassay technique. HBsAg loss was defined as two consecutive HBsAg titers < 0.05 IU/ml, measured with the Elecsys HBsAg II Quant kits (Germany). The baseline HBV load was measured by nucleic acid fluorescent quantitative polymerase chain reaction (PCR), and the chemicals were purchased from DAAN GENE (Guangzhou, China). The lower limit of HBV DNA detection was 100 IU/ml. Logarithmic transformation of quantitative HBsAg values and baseline HBV DNA values were finally obtained.

### 2.3. Definitions

Chronic hepatitis B was defined as follows: (1) HBsAg present for ≥6 months, (2) serum HBV DNA varies from undetectable to several billion IU/ml, and (3) normal or elevated ALT and/or AST levels [6]. HCC was diagnosed by at least two imaging studies (i.e., hepatic ultrasound together with CT, MRI, or both), and most cases were histopathologically confirmed according to the AASLD (2018) guidelines [6].

### 2.4. Statistical Analyses

Frequency and percentage (%) were used to describe categorical variables, median and interquartile range were used for non-parametric continuous variables. Mann-Whitney *U* test and *χ*^2^ test were used for intergroup differences. COX regression analysis was used to analyze both the univariate- and multivariate-adjusted rate ratios (with 95% confidence intervals) of HBsAg loss. Variables significant in univariate analyses were included in multivariate analyses. The statistical analysis was carried out using IBM SPSS 25.0, the *p* value was taken bilaterally, and *p* < 0.05 indicated statistically significant difference.

The nomogram was built based on the results of multivariate analyses of BMI, quantitative HBsAg and HBeAg status in the primary cohort, and baseline HBV DNA values. SAS 9.4 was used to randomly divide both the HBsAg seroclearance and nonseroclearance groups into a 1 : 2 ratio: two-thirds of cases used as training sets and the remaining one-third as validation sets. R 3.5.1 (http://www.r-project.org/) was used for constructing the nomograms with the survival using rms, grid, and ggplot2 graphics package. Nomogram validation consisted of discrimination and calibration by using the validation set. Discrimination and predictive performance of the nomogram were evaluated using a concordance index (C-index). C-index values range from 0.5 to 1.0, with 0.5 indicating random chance and 1.0 indicating a perfect ability to correctly discriminate the outcome using the nomogram [7]. *p* value < 0.05 was considered statistically significant. The calibration curve was derived based on regression analysis.

## 3. Results

### 3.1. Clinical Data Characteristics

**Figure 1:**
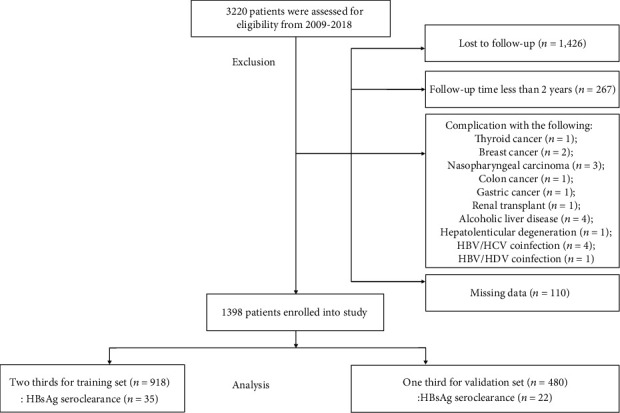
Flowchart of study.

Finally, 1398 CHB patients were enrolled in this study (Figure 1), and the cohort was divided into two sets: the training set (*n* = 918, HBsAg loss cases: 35) and validation set (*n* = 480, HBsAg loss cases: 22). After comparison between the two sets of data, there was no statistical difference regarding index variables (all the *p* values were >0.05). Long-term follow-up cohorts for the training and validation sets showed male predominance (72.77%; 77.08%), with differences found in median age (34 years; 35 years), median BMI (21.80; 22.04), report of familial history of HBV (60.57%; 61.25%), and median baseline HBV DNA (4.750 log_10_ IU/ml; 4.197 log_10_ IU/ml). AST was within the normal range (median value: 36 U/l) while ALT was mildly elevated (median value: 38 U/l) in both sets. Besides, the median follow-up time was 75 months in both sets (Table 1). In addition, the clinical features of patients with HBsAg loss are shown in Supporting Information Table S-1.

### 3.2. Independent Factors in the Training Set

The independent factors of HBsAg loss in the training set demonstrated in Table 2 were identified using univariate analysis and multivariate Cox regression analysis. Quantitative HBsAg values were subdivided into four grades (<1 log_10_ IU/ml; ≥1 log_10_ IU/ml vs. <2 log_10_ IU/ml; ≥2 log_10_ IU/ml vs. <3 log_10_ IU/ml; and ≥3 log_10_ IU/ml). The HBeAg status was set as positive or negative, and the baseline HBV DNA load was divided into four grades (<2 log_10_ IU/ml; ≥2 log_10_ IU/ml vs. <4 log_10_ IU/ml; ≥4 log_10_ IU/ml vs. <7 log_10_ IU/ml; and ≥7 log_10_ IU/ml). The risk factors for univariate analysis were BMI (*p* = 0.016), abnormal AST (≥40 U/l) (*p* = 0.027), abnormal ALT (≥35 U/l) (*p* = 0.004), baseline HBV DNA (<2 log_10_ IU/ml; ≥2 log_10_ IU/ml vs. <4 log_10_ IU/ml (*p* = 0.005); and ≥4 log_10_ IU/ml vs. <7 log_10_ IU/ml (*p* = 0.047)), quantitative HBsAg, HBeAg status, and antiviral treatment. The multivariate analysis showed that BMI (*p* = 0.001), quantitative HBsAg (≦2 log10 IU/ml;*p* < 0.001), and HBeAg status (*p* = 0.016) were independent factors of HBsAg seroclearance in CHB patients.

### 3.3. Prognostic Nomogram for HBsAg Loss of CHB

**Figure 2:**
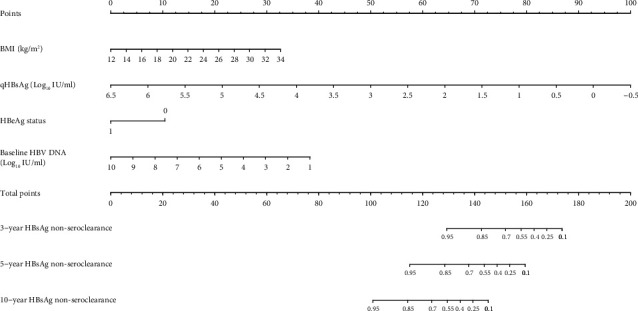
Nomogram predicting the probability of HBsAg non-seroclearance at 3, 5, and 10 years, using BMI, quantitative HBsAg (qHBsAg), HBeAg status, and baseline HBV DNA. To use the nomogram, an individual patient's value is located on each variable axis, and a line is drawn upward to determine the number of points received for each variable value. The sum of these numbers is located on the total points axis, and a line is drawn downward to the non-seroclearance axes to determine the likelihood of 3-year, 5-year, and 10-year non-seroclearance. BMI: body mass index; qHBsAg: quantitative hepatitis B virus surface antigen; HBeAg: hepatitis B virus e-antigen (0 and 1 represent negative and positive status, respectively); baseline HBV DNA: baseline hepatitis B viral deoxyribonucleic acid load.

A nomogram that incorporated the above-mentioned significant prognostic factors and baseline HBV DNA load, another important factor reported in the literature, was established (Figure 2). The prognostic nomogram for HBsAg loss of CHB patients showed that variable “quantitative HBsAg” substantially contributed to prognosis, followed by BMI, HBeAg status, and baseline HBV DNA load. Harrell's C-index for HBsAg loss prediction was 0.913 (95% CI, 0.868 to 0.958). Each subtype within the above-mentioned variables was assigned a score on the point scale. By summing up the total score and locating it on the total point scale, it was easy to draw a straight line down to determine the estimated probability of HBsAg non-seroclearance at each score point.

### 3.4. Calibration and Validation of the Nomogram

**Figure 3:**
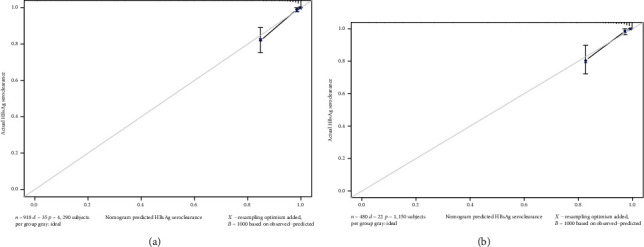
The calibration curves for predicting HBsAg seroclearance for the training cohort (a) and the validation cohort (b). Nomogram-predicted probability of HBsAg seroclearance is plotted on the *x*-axis; actual HBsAg seroclearance is plotted on the *y*-axis.

The calibration plots presented an acceptable agreement in the training and validation sets between nomogram predictions and actual observations of HBsAg loss rates (Figures 3(a) and 3(b)), and the C-index in the validation set was 0.886 (95% CI, 0.812 to 0.960).

## 4. Discussion

Despite the introduction of an effective HBV vaccine decades ago, the burden of chronic HBV infection remains a public health concern, particularly in endemic regions of Asia and sub-Saharan Africa [8]. HBsAg loss is regarded as a positive achievement in the natural history, particularly if it occurs before the accrual of significant liver disease and is deemed as a functional “cure.” However, HBsAg is infrequently cleared in CHB patients [9]. Chu and Liaw [5] reported that the predictive factors for HBsAg seroclearance can be divided into host factors including age, gender, normal alanine aminotransferase levels, and viral factors including HBeAg negativity at baseline, HBV DNA negativity by hybridization at baseline, genotype, and hepatitis C virus superinfection. A systematic review and meta-analysis of HBsAg clearance rates and predictors of clearance by Yeo et al. [10] showed that favorable factors for HBsAg loss included HBeAg seronegativity, low quantitative HBsAg values, and low HBV load at baseline. In our study, the independent risk factors of multivariate analysis were BMI, quantitative HBsAg, and HBeAg status, which is consistent with the previously reported literature. Chu et al. also reported HBsAg carriers with hepatic steatosis had significantly higher BMI at HBsAg seroclearance than those without [11, 12]. Based on such findings, it can be inferred that BMI may be a contributing factor for HBsAg seroclearance.

A nomogram is a simple graphical representation of a prediction model that generates a numerical probability of a clinical event and can be an important component of modern medical decision-making, if carefully constructed to answer a focused question and appropriately interpreted and applied [4]. It is a powerful unprecedented tool that can be harnessed to predict individual outcomes, in this case seroclearance. In the field of hepatopathies, nomograms are widely used in HCC [13] and acute on chronic liver failure (ACLF) [14]. Based on the factors mentioned above, the regression model can calculate the probability of target events in a certain period of time, such as 3, 5, and 10 years. At present, to our knowledge, there is no literature regarding any validated model that can reliably predict HBsAg loss. We envisaged the possibility of constructing a nomogram predicting HBsAg seroclearance as our clinical endpoint, incorporating factors which were proven to be independent by multivariate Cox regression analysis. Antiviral therapy for CHB patients is a long process that often leads to poor patient compliance. It has become a major conundrum for clinicians to estimate the probability of HBsAg loss and estimate length of antiviral treatment required for CHB patients. Considering HBsAg loss as the clinical endpoint of our study, we constructed a nomogram that could calculate the individual probability of seroclearance and non-seroclearance in CHB patients. In this study, we first developed and validated a new nomogram model based on BMI, HBeAg status, quantitative HBsAg, and baseline HBV DNA load to better predict the HBsAg loss rate of patients with CHB. Harrell's C-index for HBsAg loss prediction was 0.913 (95% CI, 0.868 to 0.958), showed good accuracy, and was verified by validation sets (0.886 (95% CI, 0.812 to 0.960)). To our knowledge, this is the first study to develop a nomogram for predicting HBsAg seroclearance and non-seroclearance in patients with chronic HBV infection.

Nonetheless, our nomogram is limited by the retrospective nature of data collection and other shortcomings of our study design that affect its robustness and reliability. Firstly, HBsAg seroclearance is a low probability event, and the number of patients with HBsAg loss enrolled in our study was relatively small. Secondly, both the training and validation sets came from our follow-up cohort. Indeed, the presence of an external validation group would have improved the clinical value of our nomogram. Lastly, differences in antiviral therapy regimens and the HBV genotype, which may play an important role in HBsAg seroclearance, were not considered in our study.

## Figures and Tables

**Table 1:** Demographics and clinicopathologic characteristics of chronic hepatitis B patients enrolled in the study.

	Training set (*n* = 918)		Validation set (*n* = 480)		
Variable	No. of patients	%	No. of patients	%	*p*
Gender					0.080
Male	668	72.77	370	77.08	
Age (years)	34.91 (28.96, 42.75)		35.99 (29.34, 42.68)		0.243
<40	610	66.45	307	63.96	
≥40; <60	274	29.85	156	32.50	
≥60	34	3.70	17	3.54	
BMI (kg/m^2^)	21.80 (19.82, 24.35)		22.04 (19.65, 24.69)		0.448
<18.5	120	13.07	63	13.13	
≥18.5; <25	606	66.01	305	63.54	
≥25; <30	180	19.61	108	22.50	
≥30	12	1.31	4	0.83	
History of alcohol intake	98	10.68	60	12.50	0.306
Family history of HBV	556	60.57	294	61.25	0.804
Family history of HCC	96	10.46	54	11.25	0.649
HCC					0.186
HCC on inclusion	11	1.20	8	1.67	
New-onset HCC	3	0.33	5	1.04	
AST (U/l)	36 (26, 59)		35 (26, 66)		0.726
≥15; <40	519	56.54	274	57.08	
≥40	390	42.48	204	42.50	
ALT (U/l)	38 (24, 76)		38 (25, 72)		0.917
≥3; <35	420	45.75	222	46.25	0.859
≥35	498	54.25	258	53.75	0.859
Albumin (g/l)	45.50 (43.39, 47.60)		45.30 (42.90, 47.48)		0.239
<36	35	3.81	13	2.71	0.282
Total bilirubin (*μ*mol/l)	13.84 (10.89, 18.70)		14.24 (10.33, 18.70)		0.933
>23.6	110	11.98	65	13.54	0.403
HBV DNA log_10_ (IU/ml)	4.750 (0, 6.945)		4.197 (0, 6.679)		0.118
<2	233	25.38	127	26.46	
≥2; <4	163	17.76	105	21.88	
≥4; <7	300	32.68	148	30.83	
>7	222	24.18	100	20.83	
qHBsAg log_10_ (IU/ml)	3.673 (3.200, 3.862)		3.738 (3.218, 3.888)		0.235
≤1	18	1.96	11	2.29	
>1; ≤2	30	3.27	16	3.33	
>2; ≤3	123	13.40	57	11.88	
>3	747	81.37	396	82.50	
HBeAg status					0.504
Positive	482	52.51	243	50.63	
Negative	436	47.49	237	49.38	
HBsAg loss	35	3.81	22	4.58	0.489
Antiviral treatment	666	72.55	346	72.08	0.853
Follow-up time (months)	75 (52, 90)		75 (51, 89)		0.905

Abbreviations: CHB: chronic hepatitis B; BMI: body mass index; HBV: hepatitis B virus; HCC: hepatocellular carcinoma; ALT: alanine aminotransferase; AST: aspartate aminotransferase; HBV DNA: hepatitis B viral deoxyribonucleic acid load at baseline; qHBsAg: quantitative hepatitis B virus surface antigen; HBeAg: hepatitis B virus e-antigen.

**Table 2:** Univariate analysis and Cox proportional hazards regression analysis of HBsAg seroclearance in chronic hepatitis B patients.

Variable	Univariate analysis				Multivariate analysis			
	*p*	Exp(*B*)	95% CI		*p*	Exp(*B*)	95% CI	
			LO	UP			LO	UP
Gender (male)	0.811	0.912	0.427	1.948				
Age (year)								
<40		Reference						
≥40; <60	0.853	1.209	0.163	8.992				
≥60	0.712	1.468	0.192	11.224				
BMI (kg/m^2^)	0.016	1.123	1.022	1.234	0.001	1.170	1.064	1.229
History of alcohol intake	0.050	2.292	1.001	5.251				
Family history of HBV	0.356	0.732	0.377	1.421				
Family history of HCC	0.744	1.190	0.420	3.372				
HCC								
HCC on inclusion	0.811	20.673	0.000	1.318*E*+12				
New-onset HCC	0.999	0.999	0.000	9.934*E*+11				
AST (U/l)								
≥15; <40		Reference						
≥40	0.027	2.555	1.112	5.870				
ALT (U/l)								
≥3; <35		Reference						
≥35	0.004	3.062	1.434	6.539				
Albumin (g/l)	0.114	1.083	0.981	1.196				
Total bilirubin (*μ*mol/l)	0.448	1.003	0.996	1.010				
HBV DNA log_10_ (IU/ml)								
<2		Reference						
≥2; <4	0.005	17.720	2.391	131.323				
≥4; < 7	0.047	8.342	1.026	67.851				
>7	0.424	2.447	0.273	21.908				
qHBsAg log_10_ (IU/ml)								
≤1		Reference				Reference		
>1; ≤2	<0.001	130.255	57.677	294.161	<0.001	106.778	43.537	261.883
>2; ≤3	<0.001	11.476	3.965	33.213	0.001	5.945	1.993	17.736
>3	0.135	2.395	0.762	7.533	0.139	2.382	0.755	7.517
HBeAg status	<0.001	11.042	3.381	36.066	0.016	4.644	1.336	16.138
Antiviral treatment	0.032	0.480	0.245	0.939				

Abbreviations: CHB: chronic hepatitis B; BMI: body mass index; HBV: hepatitis B virus; HCC: hepatocellular carcinoma; ALT: alanine aminotransferase; AST: aspartate aminotransferase; HBV DNA: hepatitis B viral deoxyribonucleic acid load at baseline; qHBsAg: quantitative hepatitis B surface antigen; HBeAg: hepatitis B e-antigen.

## Data Availability

The data used to support the findings of this study are available from the corresponding author upon request.

## Abbreviations

CHB: Chronic hepatitis B
HBV: Hepatitis B virus
HBsAg: Hepatitis B virus surface antigen
HBeAg: Hepatitis B virus E antigen
HBsAb: Hepatitis B virus surface antibody
HBeAb: Hepatitis B virus E antibody
HBcAb: Hepatitis B virus core antibody
HCC: Hepatocellular carcinoma
ACLF: Acute on chronic liver failure
ALT: Alanine aminotransferase
AST: Aspartate aminotransferase
ALB: Albumin
BMI: Body mass index
PCR: Polymerase chain reaction.

## Supplementary Materials

Supplementary Materials Table S-1: a summary of the clinical features of all patients which attained HBsAg seroclearance in our study.
