# Supplementary material for: Prediction Model of HBsAg Seroclearance in Patients with Chronic HBV Infection
Source: Biomed Res Int. 2020 Aug 14;2020:6820179. doi: 10.1155/2020/6820179 (PMC7443222; doi:10.1155/2020/6820179)
Supplement: Supplementary Materials — Table S-1: a summary of the clinical features of all patients which attained HBsAg seroclearance in our study. [file 6820179.f1.pdf]

Table S-1. Clinical features of CHB patients with HBsAg seroclearance.

Variable		No. of patients (%)/ median (interquartile range)
Gender		
	Male	47 (82.46)
	Female	10 (17.54)
Age (years)		37.42（30.20, 46.09）
BMI (kg/m^2)		22.96（19.88, 26.23）
History of alcohol intake		
	Yes	11 (19.30)
Family history of HBV		
	Yes	27 (47.37)
Family history of HCC		
	Yes	5 (8.77)
AST (U/L)		32.00 (24.00, 45.50)
ALT (U/L)		29.00 (20.00, 47.50)
Albumin (g/L)		46.70 (44.55, 48.40)
HBV DNA log_{10} (IU/ml)		0（0, 3.33）
qHBsAg log_{10} (IU/ml)		1.76 (0.39, 3.22)
HBeAg status		
	Positive	7 (12.28)
	Negative	50 (87.72)
Antiviral treatment		33 (57.89)
	Nucleoside/nucleotide analogue	28 (49.12)
	Interferon	2 (3.51)
	Nucleoside/nucleotide analogue+Interferon	3 (5.26)
No antiviral treatment		24 (42.11)
HBeAg clearance		2 (3.51)
HBeAg seroconversion		10 (17.54)
Follow-up time (months)		56.83 (35.60, 74.10)

Abbreviations: CHB: Chronic Hepatitis B; BMI: Body Mass Index; HBV: Hepatitis B Virus; HCC: Hepatocellular Carcinoma; ALT: alanine aminotransferase; AST: aspartate aminotran-

sferase; HBV DNA: Hepatitis B Viral Deoxyribonucleic acid load at baseline; qHBsAg: quantitative hepatitis B surface antigen; HBeAg: Hepatitis B e-antigen.
